# Teeth, Pregnancy and Disease

**Published:** 1880-01

**Authors:** Basil M. Wilkerson

**Affiliations:** Baltimore


ARTICLE VII.

### TEETH, PREGNANCY AND DISEASE.

BY BASIL M. WILKERSON, D. D. S., M. D., BALTIMORE.

It is not my object here to take up and treat individual disease and its peculiar effects upon the teeth. But if this short article should cause the medical and dental practitioner to think of the duties they reciprocally owe each other during times of peril the world will be happier.

Much devolves on the physician and dentist in the care of the teeth during pregnancy, and should the former have the opportunity it is his duty to advise his patient to have her teeth put into perfect condition before she becomes pregnant. He is well aware of the close sympathy between the teeth and the uterus, as well as their complications. If she acts on his advice, and secures the professional services of a dentist who is apprized of the fact, and comprehends the importance and result of his operations, she will be far less susceptible to odontalgia and other algias. When dental operations are to be performed for pregnant women, the dentist had best confer with her physician.

Under whose care do the teeth first fall? That of the medical practitioner. He should administer to the mother such food or medicines as contain the proper elements to nourish the dental papillæ while yet in utero. With such treatment the dental arch will be better developed and a more durable class of teeth born to the nation. They, though not yet visible, must not be deprived of the required nutrition. Teeth having had this care are more likely to be possessed by a healthy child; and when they reach the dentist, he is not brought into contact with a bundle of nerves which repel all of his kind efforts and persuasions, but can operate with a firm hand on solid material, and with confident hope of success.

During disease of the body the teeth have often to stand the most severe ordeal. It is then that they are neglected almost entirely, and that the most deleterious agents are brought into contact with them. The remedy is to keep them clean, and the fluids, as they flow into the mouth, non-corrosive by artificial means; not to allow acid or alkaline medicines to come in contact with them but when it is unavoidable, and use a neutralizer immediately. These precautions should always be adhered to, except in cases of extreme illness, when it might endanger life to do so. At the return of a patient, after a spell of sickness, a faithful dentist has been made to blush at the condition in which he finds his operations.

The physician should care for those teeth that be and are to be, and the dentist for those that "be or not to be."

In the foregoing we have offered nothing new, but it is hoped that the hints will cause practitioners to *think*, *act*, *write* and enlarge on this *very important* subject. Perhaps more should be said now, but time and space admonish a conclusion.

